# Liquid-liquid extraction of selenium (IV) ions from hydrochloric acid solution using Aliquat 336 dissolved in kerosene

**DOI:** 10.1186/s13065-024-01288-y

**Published:** 2024-09-28

**Authors:** Mohamed I. Aly, S. E. Rizk

**Affiliations:** Hot Laboratories Center, Egyptian Atomic Energy Authority, Cairo, 13759 Egypt

**Keywords:** Selenium(IV), Aliquat 336, Hydrochloric acid, Solvent extraction

## Abstract

Solvent extraction of selenium(IV) ions from highly concentrated hydrochloric acid using 0.4 mol/L Aliquat 336 dissolved in kerosene was investigated. As a modifying agent, 1-octanol (10% v/v) was added to the organic phase to avoid the third phase formation. The effect of different parameters affecting the liquid-liquid extraction of selenium(IV) such as the acid concentration, shaking time, metal ion concentration in the aqueous phase, loading capacity, diluents, and temperature, was studied. The results indicate that selenium(IV) is extracted efficiently by 0.4 mol/L Aliquat 336 dissolved in kerosene. It was noticed that the extraction increased with the increase in the acid and Aliquat 336 concentrations, reaching an extraction percentage of about 92% at 8 mol/L HCl and 97.1% at 1 mol/L extractant. The extracted organic species is postulated to be [H_2_SeO_2_Cl_2_.2R_4_NCl]_org_ by using the slope analysis method, and the value of K_ex_ for selenium(IV) extraction was found to be 26.17 ± 2 M^− 2^. The structure of the extracted organic species was confirmed by FT-IR. The effect of diluents using various aliphatic and aromatic diluents indicated that kerosene is the most preferred diluent. This is owing to safety ground purpose, economic consideration, the lower cost, availability, and lower toxicity. Thermodynamic parameters indicate the endothermic nature for the solvent extraction of selenium(IV) for the investigated system according to the positive value obtained of the enthalpy change (ΔH). Depending on the obtained results, the method was used to recover selenium(IV) from a simulated solution synthesized in hydrochloric acid medium, which is expected in anode slime leach liquor solution.

## Introduction

Selenium is an element that has sparked scientific concern across a wide range of fields, such as medical and industrial scales. Selenium has various significant applications in the chemical and metal alloy industries, electronics (as it has good photoconductive and photovoltaic properties), medicine (owing to its antioxidant properties), and the production of semiconductor products [1]. Additionally, selenium is used extensively in the production of glass, ceramics, and the rubber industry [2–5]. One of the radioactive isotopes of selenium, selenium-75, is utilized in studying the functions of the pancreas and parathyroid gland. Selenium is considered a metalloid element that can be found in four chemical oxidation states: in the solid phase, it exists as the native element Se(0); in minerals, it is selenide (Se(-II)); in the aqueous phase, selenite (Se(IV)) or selenate (Se(VI)) exist as SeO_3_^2−^ and SeO_4_^2−^ species, respectively [6]. There are seven naturally occurring isotopes of selenium. Five of them– ^74^Se, ^76^Se, ^77^Se, ^78^Se, and ^80^Se–are stable. The latter three occur as fission products, along with ^79^Se, which also occurs in trace amounts in uranium ores as a product of nuclear fission [7–9].

Liquid–liquid extraction is the most widely used method for extraction and purification of different metals on both a laboratory and industrial scale, including hydrometallurgy and nuclear fuel reprocessing [10, 11]. Liquid-liquid extraction means either solvent extraction or partitioning. Solvent extraction separation depends mainly on the difference in solubilities of elements and their compounds in two immiscible liquid phases. It was considered a transfer of a substance (metal ion) from one liquid phase (feed or source phase) into another immiscible liquid phase (solvent or extract phase) based on differences in their solubilities or affinities for the solute [12–15]. This method holds a privileged position among methods of separation in industry according to its efficiency, selectivity, sensitivity, and versatility [12–15]. Solvent extraction is a versatile technique used to extract a wide range of metals, including rare earth elements (REEs) [16], uranium [17], base metals (like copper, zinc, nickel, and cobalt) [18], other metals like lithium, molybdenum, and even some precious metals [18].

Ionic liquids (ILs) are currently the subject of intense investigations since they provide safe alternatives to the common molecular solvents and are friendly to the environment. These ionic liquids are called the green organic solvents due to their low vapor pressure, low volatility, and adjustable functional group in addition to their non-flammability and non-toxicity nature [19]. Hydrophobic Aliquat 336 is one of the ionic liquids that is a long–chain quaternary ammonium salt with the common chemical formula R_4_N^+^, where R is an alkyl group with eight or ten carbon atoms. As a result of the increasing usage of Aliquat 336 as an extractant for the removal of metal ions from industrial waste, the capability of this versatile extractant for selenium needs to be investigated. While ammonium salts have been traditionally used, quaternary phosphonium and pyridinium salts hold promise as viable alternatives for selenium(IV) extraction due to their potential for improved selectivity, stability, and reusability. The non-functional ionic liquids (ILs) such as tricapryl methyl ammonium chloride and trihexyl (tetradecyl) phosphonium have been widely used in solvent extraction for the recovery and separation of metal ions [20–22].

There aren’t many selenium(IV) extraction techniques documented in the literature. However, several studies have reported on the investigations of selenium using different extractants. Sargar et al. [3] studied simple quantitative and selective extraction of selenium(IV) by N-n-octylaniline in xylene from 0.4 to 0.6 mol/L hydrochloric acid solution. They found that selenium(IV) was extracted into the organic phase by ion-pair formation complex as [RR^/^NH^+^ _2_HSeO^−^_3_]_org_. This method is efficient for the determination of selenium(IV) in synthetic mixtures. Sattari et al. [23] studied the extraction of selenium in HCl media using triisobutyl phosphate (TIBP) and a triisobutyl Phosphate/ dodecanol mixture. They found that the extraction of selenium(IV) was better with the organic phase containing 20 vol% TIBP diluted with kerosene and at ambient temperature. Additionally, using dodecanol as a modifier has an enhancement impact as it increases selenium extraction. Mhaske et al. [24] studied the extraction and separation of selenium(IV) and tellurium(IV) from aqueous hydrochloric acid solution using Cyanex 925 dissolved in toluene. The authors found that the facilitation of separation of these metal ions is related to the variation in their extractability toward Cyanex 925 from the hydrochloric acid medium. Jagatap et al. [5] investigate a sequential mode for selenium(IV) and Te(IV) extraction using N-n-octylcyclohexylamine (N-n-OCA) in a dichloromethane (DCM)–xylene mixture from HCl solution. The authors found that N- n- OCA extracts these metal ions in DCM - xylene from hydrochloric acid media by anion exchange mechanism, and the extracted species [RR’NH_2_ + HSeO_3_^−^] and [(RR’NH_2_^+^)2TeCl_6_^2−^] are formed. Sasaki et al. [25] studied the solvent extraction of elements including long-lived radionuclides as Cs(I), Zr(IV), Se(IV), and Pd(II) from nitric acid with 1-octanol or 1-octanol and n-dodecane. It was found that over 90% extraction of these elements was obtained. The increase in HNO3 concentration increases the recovery of Cs(I) and Zr(IV) due to ion-pair extraction while decreasing the recovery of Pd(II) and Se(IV) due to protonation of extractants. Chowdhury et al. [26] studied the diluents effect on the extraction of Se(IV) and Te(IV) by 5 and 30 vol% tributyl phosphate (TBP) from 4.5 mol/L aqueous hydrochloric acid. The authors observed a decrease in the distribution coefficient of both metals with the increase in the non-polar diluent solubility parameter. Kerosene and hexane are competitive with one another in terms of distribution coefficient. Kerosene costs less than hexane, but it is more likely to produce a third phase for metal salts and concentrated hydrochloric acid. Additionally, Chowdhury et al. [4] studied the extraction and separation of selenium(IV) from tellurium(IV) with 1.1 mol/L TBP dissolved in kerosene as an extractant. It was observed that a 10 mol/L initial acid concentration was efficient for the extraction of selenium(IV). The metal-extracted species were found to be SeOCl_2_(TBP)_2_ and TeCl_4_(TBP)_3_ at 3–5 mol/L HCl and H_2_SeOCl_4_(TBP)_2_ at 10 mol/L HCl.

Mahmoudiani et al. [27] used the solvent extraction method to study the separation of selenium(IV) and Te(IV) from HNO_3_ medium using CYANEX 301 dissolved in kerosene. They found that a separation factor of 201.7 has been achieved, and the extraction of selenium was higher than 98%. Also, complete recovery of selenium(IV) was carried out after three stages of stripping using a mixture of 0.06 mol/L KSCN and 2.0 mol/L HNO_3_.

Pasdar et al. [28] investigated the separation of selenium from copper anode slime, which consists mainly of gold, silver, selenium, and tellurium. They reported that the optimum conditions for selenium recovery by alkaline roasting were at a temperature of 600 ^O^C and a time of 8 h. They indicated that about 98% selenium was recovered by the mentioned method.

In this concern, the current work focuses on the liquid-liquid extraction of selenium(IV) from hydrochloric acid solution using the hydrophobic Aliquat 336 dissolved in kerosene diluent with the addition of 1-octanol to prevent the third phase formation. The different factors affecting the extraction behavior of selenium(IV) were investigated. The investigated parameters were the metal ion concentration, acidity effect, extractant concentration, loading capacity, O/A phase ratio, and temperature in order to get the optimum conditions for the recovery of selenium(IV) from an aqueous acidic medium. The mechanism of extraction and the nature of the extracted complex were also sought. The stripping investigations on the organic phase loaded with selenium(IV) were performed using different stripping agents. A simulated solution of the leach liquor of copper anode slime was synthesized. The extractability of selenium under studied conditions was achieved efficiently.

## Experimental

### Reagents

All the chemicals used in this experiment were analytical reagent (AR) grade. Hydrochloric, sulfuric, and nitric acids are Merck products (Germany). Sodium hydroxide was supplied from Aldrich (Germany). SeO_2_ was procured from Merck (Germany). Aqueous solutions, which were prepared from selenium and stock solutions of SeO_2_ in hydrochloric acid, were stored in well-sealed flasks to minimize air oxidation. Aqueous solutions in concentrated hydrochloric acid used double distilled water to dissolve the metal ions and prepare the aqueous solution of this study. Aliquat 336 [29], supplied from Merck (England), is composed of a tricaprylylmethylammonium chloride and trioctylmethylammonium chloride mixture of C_8_ and C_10_ chains with C_8_ predominating. It is used as a metal extraction reagent and a phase-transfer catalyst with a chemical structure as shown below [30]. Generally, Aliquat 336 is a water-insoluble quaternary ammonium salt composed of a large organic cation associated with a chloride ion. The non-aromatic, odorless kerosene was purchased from the Egyptian company Misr Petroleum.

**Figure:**
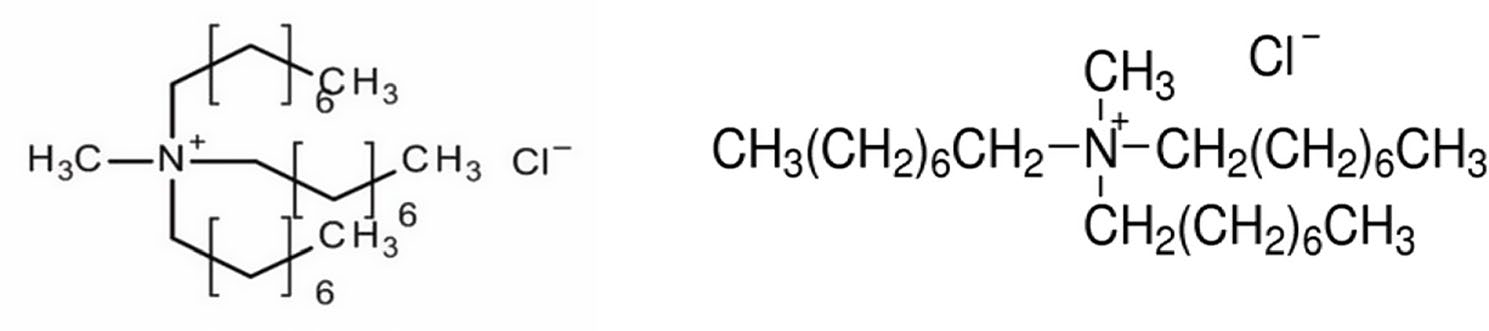
Chemical structure of Aliquat336 mentioned in [29, 30]

### Equipment

Selenium(IV) in the aqueous phase was determined by an atomic absorption spectrophotometer, (model S4 series, thermo–electron corporation) [31]. A thermostatic water bath shaker was used to mix an equal volume of organic and aqueous phases at a temperature of 25 ± 1 °C for 45 min, which was sufficient to attain equilibrium (increasing the shaking did not affect the findings of the extraction tests). The difference in metal concentration between the aqueous phase before and after extraction was used to compute its concentration in the organic phase and was checked from time to time by scrubbing and stripping a sample of organic phase with 0.5 mol/L and 6 mol/L hydrochloric acid solution, respectively, and analyzing it in the same way as for the aqueous sample. FT-IR spectrometer 6300 (from JASCO Corporation in Tokyo, Japan) was used to perform FT-IR of the commercial Aliquat 336 in kerosene and their complexes with selenium(IV) along the range 400–4000 cm^− 1^ during the IR investigations. The agilent inductively coupled plasma-optical emission spectrometer, the ICP-OES 5800 (California, USA), is used in the determination of the mixture sample.

### Extraction experiments

Extraction of selenium(IV) by Aliquat 336 experiments was conducted by mixing equal volumes of organic phase of 0.4 mol/L Aliquat 336/kerosene in the presence of 10% octanol to prevent the creation of a third phase with an aqueous phase of 0.05 g/L selenium(IV) in 6 mol/L hydrochloric acid solution in tightly closed stoppered glass bottles using a thermostatic shaking water bath adjusted at 25 ± 1^o^C. After equilibration and phase separation, a suitable dilution of the aqueous phase was done in order to analyze selenium(IV) by atomic absorption spectrophotometer.

Selenium(IV) concentration in the organic phase was investigated by the mass balance of selenium before and after the extraction. The equilibrium distribution coefficient (D) of selenium(IV) ions between aqueous and organic phases was estimated from Eq. 1:

1 $$\:D=\:\frac{({C}_{o}-C)}{C}$$

where, C_o_ and C are the selenium(IV) concentrations in the aqueous phase before and after the extraction, respectively. The extraction percentage (%E) is estimated from Eq. 2:

2 $$\:\%E=\:\frac{100\:D}{(D+1)}$$

In the stripping investigation, the loaded organic layer was conducted with an equal volume of stripper solution under test for one hour at 25 *±* 1^o^C. The stripping percentage (% Strip.) was given by Eq. 3:

3 $$\:\%Strip=\left[\:\frac{{C}_{s}}{\left({C}_{o}-C\right)}\:\right]\times\:100$$

where, C_s_ is the metal ion concentration in the aqueous phase after stripping.

### Application investigations

The examination of selenium(IV) dissolved in HCl (aqueous phase)/Aliquat 336 dissolved in kerosene (organic phase) extraction system at optimum conditions has been studied. This investigation has taken place on selenium extraction from interfering ions that may be found in solution of the leach liquor of copper anode slime. The analysis of selenium(IV) and other associated metal concentrations in synthetic leaching solution containing 0.05 g/L of each metal ion [Se(IV), Ag(I), Cu(II), Au(III), and Te(IV)] in 6 mol/L HCl was carried out using ICP-OES 5800.

## Results and discussion

### Extractant screening

Different extractants dissolved in kerosene were preliminary investigated for the extraction of selenium(IV) from high acidic solution (6 mol/L) under the same experimental circumstances (O/A = 1:1, shaking time = 30 min, and 25 ± 1 ^o^C). The examined extractants are either organic extractants (neutral as CYANEX925 or acidic HDEHP and HTTA) or ionic liquids as Aliqaut 336. The obtained results revealed that the extraction efficiency of the investigated extractants gradually declined in the order: Aliqaut 336 > CYANEX925 > HDEHP > HTTA. Therefore, according to the observed extraction efficiency, Aliqaut 336 was selected for our study as it gives the higher extraction percentage (%E) for selenium (reached about 87.3%) than the other examined extractants (%E = 28.04%, 70.1%, and 36.4% for HTTA, CYANEX 925, and HDEHP, respectively). Furthermore, Aliqaut 336 is considered an example of green chemistry compared with the traditional extractants. Additionally, to yet, no information is available to evaluate the impact of this exposure.

### Solvent extraction of selenium(IV)

#### Impact of shaking duration

**Fig. 1:**
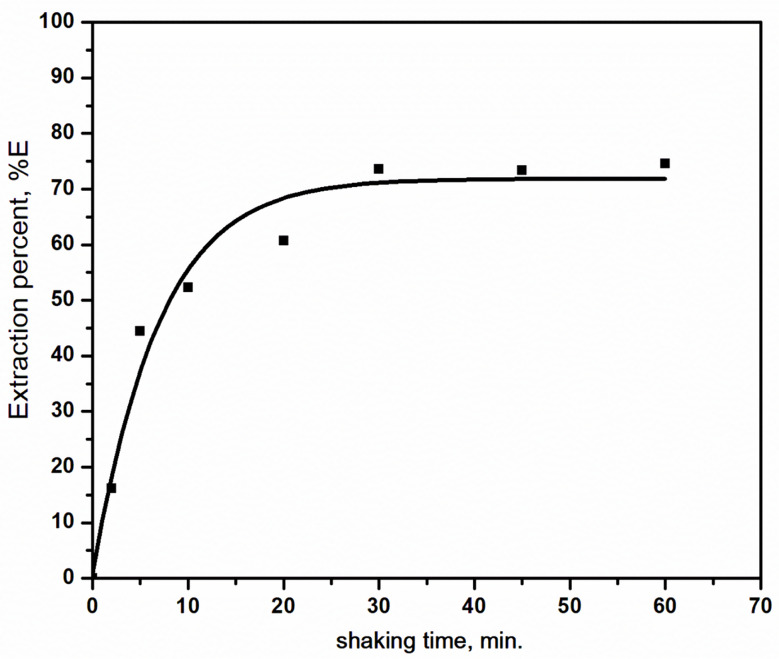
Effect of shaking time of the extraction of selenium(IV) using Aliquat 336 dissolved in kerosene. [Aliquat 336] = 0.4 mol/L; [HCl] = 6 mol/L; [Se(IV)] = 0.05 g/L; T = 25 ^o^C; O:A = 1:1

For the extraction of 6.3 × 10^− 4^ mol/L selenium(IV) with 0.4 mol/L Aliqaut 336 in kerosene-based solution, the influence of shaking duration was examined in the range of 2–60 min, as shown in Fig. 1. It was observed that the extraction of selenium(IV) increased with longer shaking times up to 30 min., plateauing at about 70% with little variation during that period of time. Therefore, an equilibration time of 30 min was sufficient for extraction equilibrium. However, all the extraction parameter experiments occurred at 45 min to ensure complete equilibrium.

#### Acid concentration’s effect

**Fig. 2:**
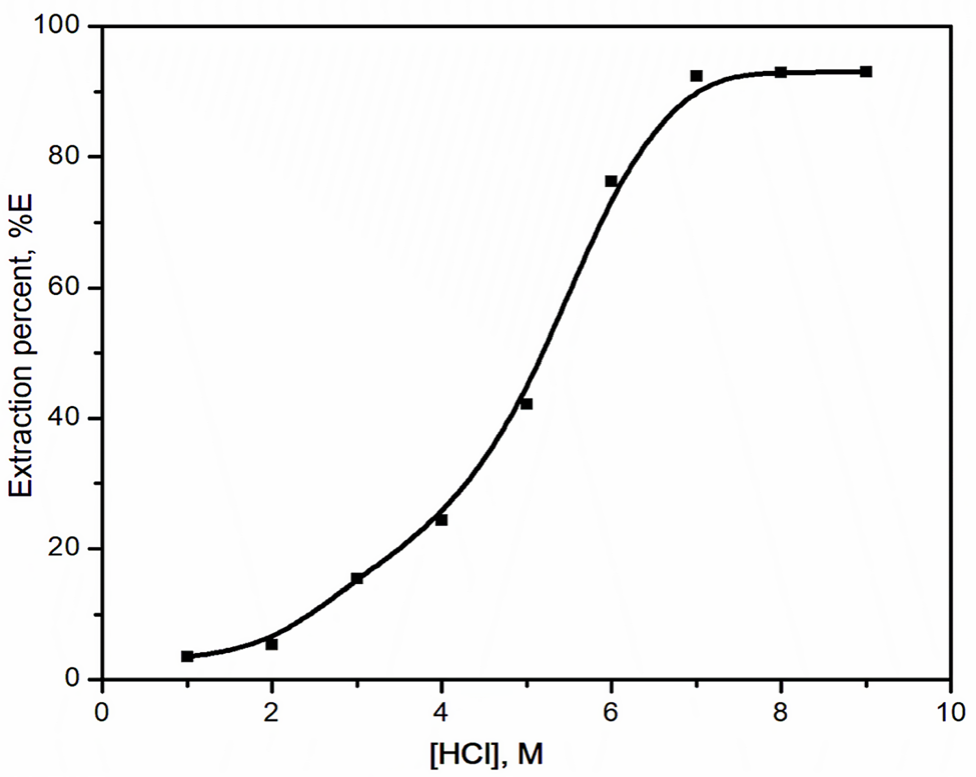
Effect of hydrochloric acid concentration on the extraction of selenium(IV) using Aliquat 336 dissolved in kerosene. [Aliquat 336] = 0.4 mol/L; t = 45 min; [Se(IV)] = 0.05 g/L; T = 25 ^o^C; O: A = 1:1

The investigation of selenium(IV) extraction was conducted in the acid range (1–9 mol/L) with 0.4 mol/L Aliqaut 336 in kerosene-based solution at a fixed concentration of 6.3 × 10^− 4^ mol/L selenium(IV). The observed extraction efficiency increased with increasing HCl concentration, nearing 92% at 8 mol/L, as can be demonstrated in Fig. 2. This reveals that at higher acid concentrations the extractability of selenium(IV) is independent of the hydrogen ion concentration, as mentioned in literature [24]. The increase in selenium(IV) extraction with rising acidity is distinctly related to decreasing water activity and a decrease in proton hydration, partially as a result of low water activity in the aqueous phase and partially as a result of acid replacing water. Additionally, the steep spike in the selenium(IV) extractability with increasing concentrations of hydrochloric acid is essentially ascribed to a substantial increase in the activity of the chloride ion in the aqueous phase [32]. At higher HCl concentrations, selenium(IV) extraction efficiency increased as a high chloride concentration in the aqueous phase increased the selenium(IV) extraction by promoting Se-Cl complex formation. Suggesting that the chloride ion participate in the selenium(IV) extraction. Consequently, it can be concluded that although the formation of selenium(IV) extractable complexes is independent of the hydrogen ion concentration, extraction is only possible in the presence of acid [33].

On the other hand, generally ammonium salts like Aliquat 336 cannot by themselves extract inorganic acids (e.g., HCl) [34–36]. The HCl co-extraction did not affect selenium(IV) extraction efficiency by Aliquat 336 as mentioned in literature [34]. This is confirmed by Le et al., indicating that aliquat 336 has no affinity to extract any hydrogen ions owing to its natural structural properties, that is, an ammonium salt [34]. Therefore, there is no competition of HC1 during extraction of selenium(IV). Subsequently, the selenium(IV) extractability in this system is not affected by an increased competition of HC1 at high HC1 concentrations [33, 37], which explains why the selenium(IV) extraction efficiency increases with a rise in hydrochloric acid concentration.

#### Effect of extractant concentration

**Fig. 3:**
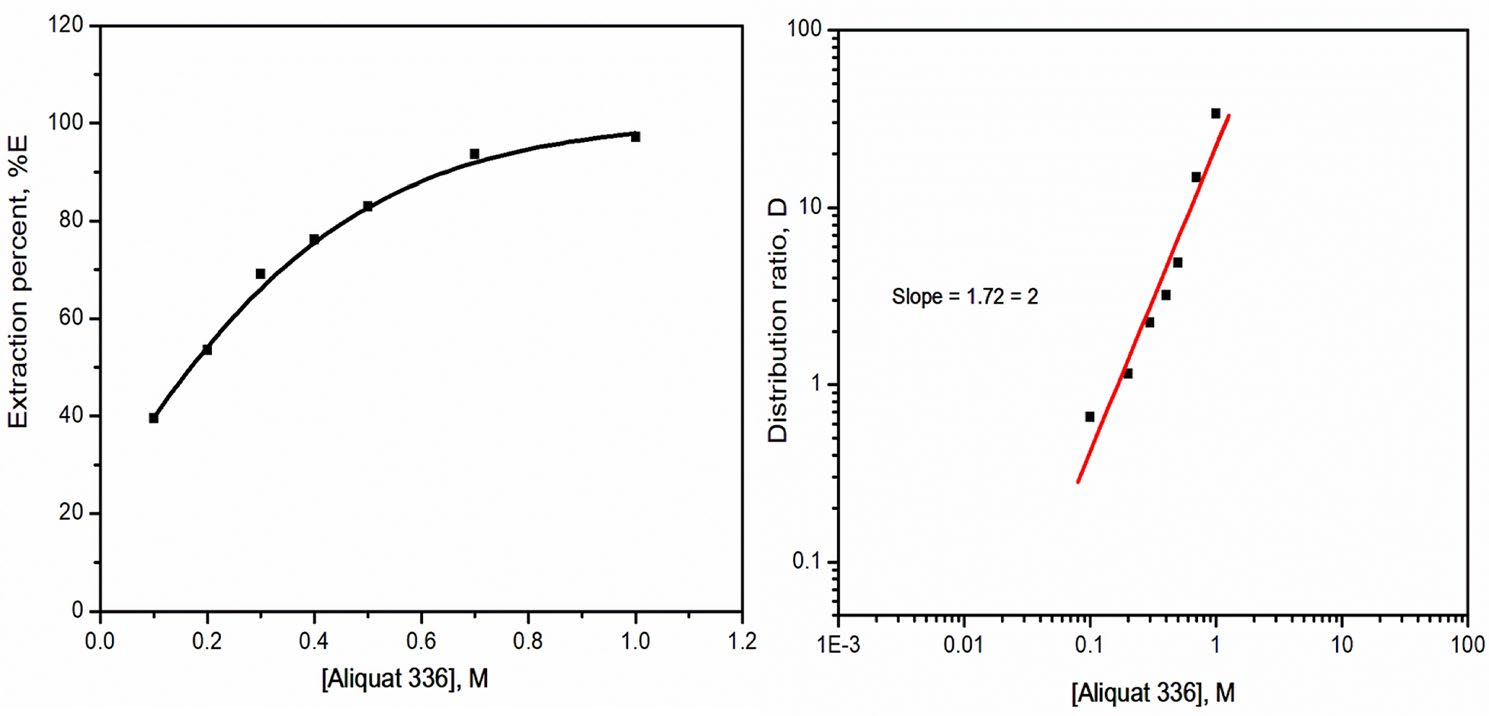
Effect Aliquat 336 concentration upon the extraction of selenium(IV) from hydrochloric acid medium. [HCl] = 6 mol/L; t = 45 min; [Se(IV)] = 0.05 g/L; T = 25 ^o^C; O: A = 1:1

The variation effect of the extractant concentration (0.1–1 mol/L) on the extraction of 6.3 × 10^− 4^ mol/L selenium(IV) was investigated. In the extractant concentration range from 0.1 mol/L to 1 mol/L, Aliqaut 336 was observed to boost the extraction efficiency. The plotting of the log-log scale between the extractant concentrations versus the distribution ratios gives a linear relation with slope equal 2, Fig. 3, indicating that there were two Aliqaut 336 molecules participating in the extracted species.

#### Effect of initial selenium(IV) concentration

**Fig. 4:**
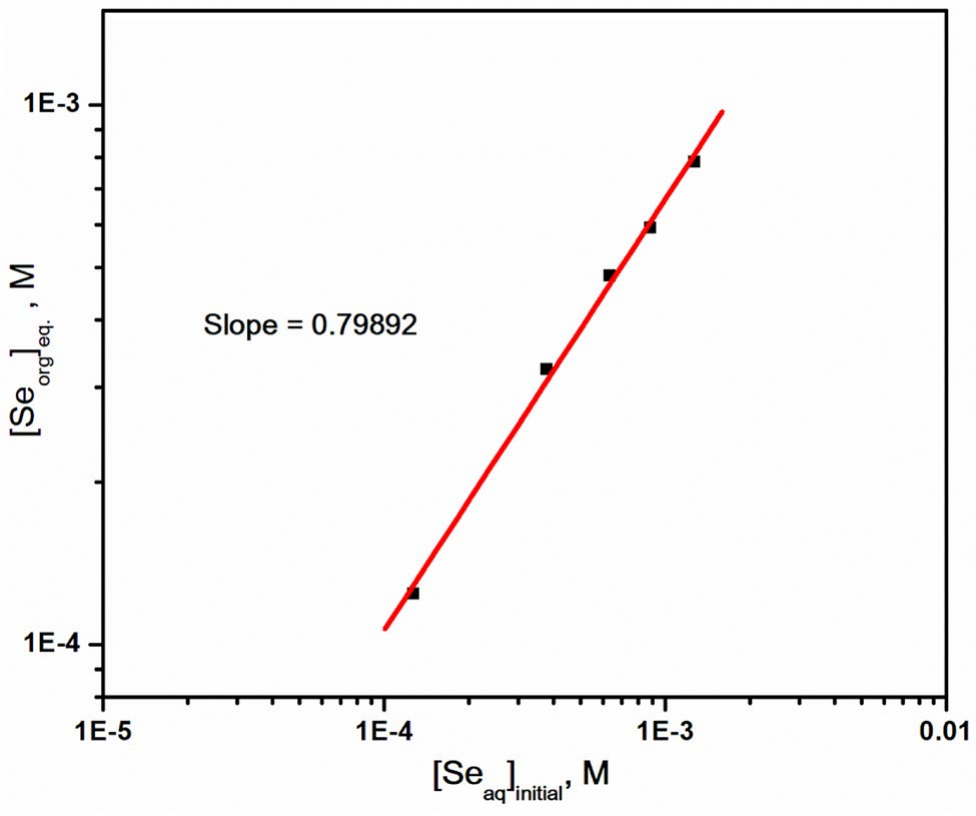
The relation between initial selenium(IV) concentration in the aqueous phase and its concentration in the organic phase at equilibrium during its extraction with Aliquat 336 / kerosene from hydrochloric acid solution. [Aliquat 336] = 0.4 mol/L; t = 45 min; [HCl] = 6 mol/L; T = 25 ^o^C; O: A = 1:1

The effect of the initial selenium(IV) ion concentration in the range 1.3 × 10^− 4^–1.3 × 10^− 3^ mol/L (as it occurs in trace amounts in uranium ores as a product of nuclear fission) on its extraction with 0.4 mol/L Aliquat 336 in kerosene from hydrochloric acid solution (6 mol/L) was investigated. Figure 4 describes the relation between the various initial selenium concentrations in the aqueous phase [Se(IV) _aq_]_initial_ and their concentrations in the organic phase at equilibrium [Se(IV)_org_]_eq_. as a log-log relation. It was found from results data that the equilibrium concentration of selenium(IV) in the organic phase increased with its concentration in the aqueous phase through a linear relation with a slope equal to 1, confirming the extraction of mononuclear species of selenium(IV) into the organic phase. This result indicates the formation of a 1:2 ratio of metal to extractant complex, which supports the results represented in Fig. 3.

#### Extraction equilibrium of selenium(IV)

The slope analysis method has been used to ascertain the nature of selenium species in the extracted system. Generally, the extraction process by quaternary ammonium salts, Aliquat 336, has occurred through an anionic exchange mechanism related to the reaction between the anionic species of the metal ions and the chloride ions of Aliquat 336, as mentioned by Liu et al. [38]. Hence the following equilibrium may be proposed by considering the SeO_3_^2−^ as an anionic species of selenium as an anion exchange mechanism.

4 $$\:Se{O}_{3}^{2-}+2{\left[{R}_{4}{N}^{+}{Cl}^{-}\right]}_{org}\:\leftrightarrow\:{\left[{\left({R}_{4}N\right)}_{2}\:{SeO}_{3}\right]}_{org}+\:2{Cl}^{-}$$

Additionally, Aliquat 336 as an ionic liquid can be used for the extraction of either neutral or charged metal species from aqueous media, relying on the extracted complex, kind of metal ion, and nature of the media [39]. According to literature Se(OH)_2_CI_2_ and H_2_SeO_2_CI_2_ were the proposed extractable species for selenium(IV) extracted from a strong hydrochloric acid medium [40]. Subsequently, the equilibrium reaction can be represented as Eq. (5), in which selenium ions are extracted by addition reactions with Aliquat 336, which agree with that given in literature [41, 42]:

5 $$\:{H}_{2}Se{O}_{2}{Cl}_{2}+2{\left[{R}_{4}{N}^{+}{Cl}^{-}\right]}_{org}\:\leftrightarrow\:{\left[{H}_{2}Se{O}_{2}{Cl}_{2}.2{R}_{4}NCl\right]}_{org}$$

**Fig. 5:**
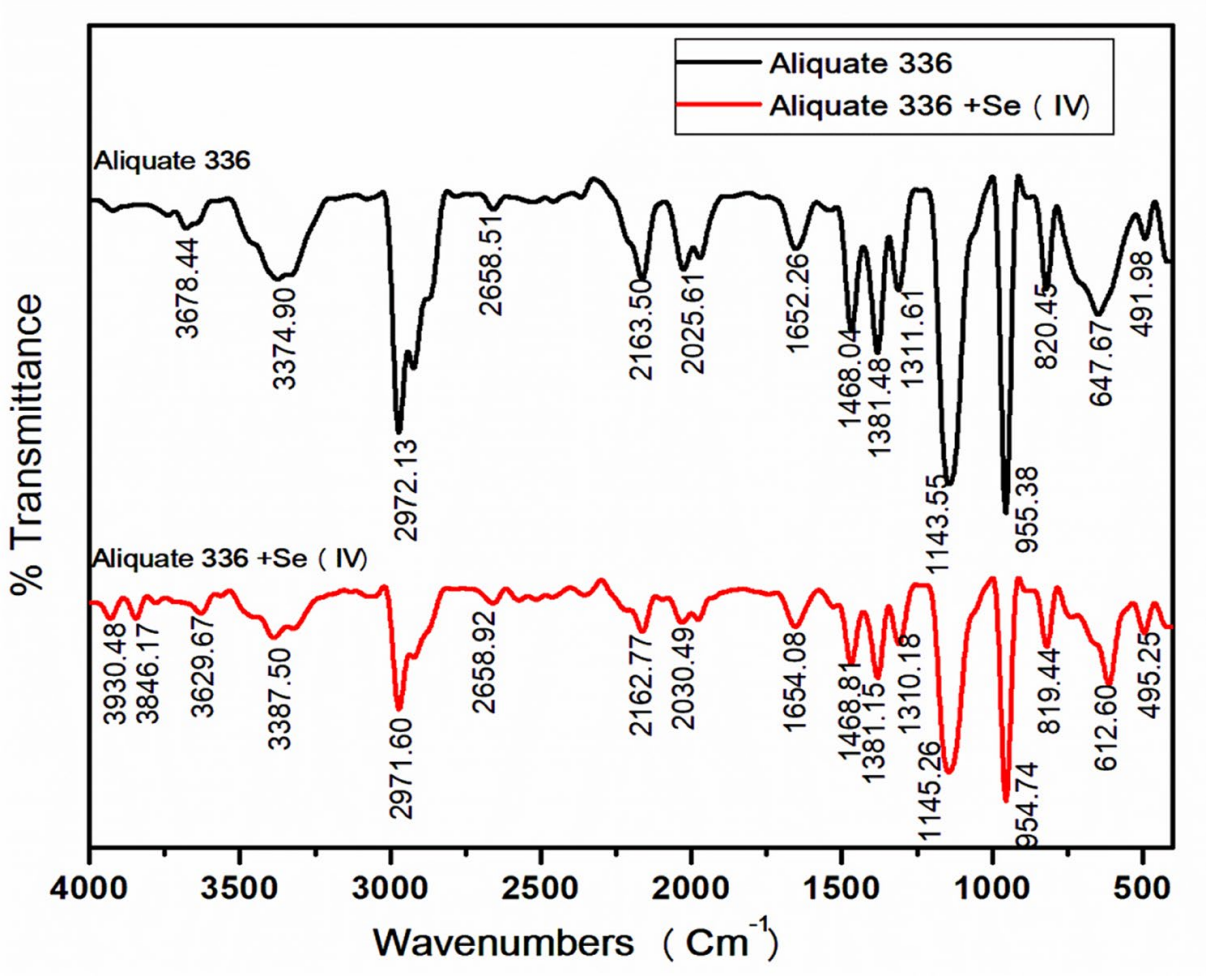
FT-IR characterizations of selenium(IV) complex extracted by 0.4 mol/L Aliquat 336 in kerosene

This assumption is confirmed by IR analysis, Fig. 5, where there is no absence or presence of new peaks for the loaded extractant with selenium(IV), but the only change is in the strength of peaks that were observed. Where the strength of significant peaks at 1468 and 1145 Cm^− 1^ for the loaded extractant was less than that of pure Aliquat 336. [H_2_SeO_2_Cl_2_.2R_4_NCl]_org_.

where *R*_4_*N*^+^*Cl*^-^ refers to Aliquat 336.The equilibrium constant is given as follows:

6 $$\:{K}_{ex}=\:\frac{{\left[{H}_{2}Se{O}_{2}{Cl}_{2}0.2{R}_{4}NCl\right]}_{org}}{\left[{H}_{2}Se{O}_{2}{Cl}_{2}\right]\:{\left[{R}_{4}{N}^{+}{Cl}^{-}\right]}_{org}^{2}}$$

7 $$\:{K}_{ex}=\:\frac{D}{\:{\left[{R}_{4}{N}^{+}{Cl}^{-}\right]}_{org}^{2}},\:\:\:\:{M}^{-2}$$

where the distribution ratio is expressed by:

8 $$\:D=\:\frac{{\left[{H}_{2}Se{O}_{2}{Cl}_{2}.2{R}_{4}NCl\right]}_{org}}{\left[{H}_{2}Se{O}_{2}{Cl}_{2}\right]\:}$$

The value of K_ex_ at different [Aliquat 336] was calculated using Eq. (7), and the average value of K_ex_ for selenium(IV) extraction using Aliquat 336/kerosene was found to be 26.17 ± 2 M^− 2^.

#### FT-IR investigations

For more clarification of the mechanism of selenium extraction, the infrared analysis spectra for the functionalized Aliquat 336 were investigated. The FTIR spectra of Aliquat 336 before and after loading with selenium(IV) were studied in the wavelength region 4000 –450 cm^− 1^ as represented in Fig. 5 to confirm the conclusions of the current experiment. The spectra indicated stretching absorption bands between 3000 and 2850 cm^− 1^ corresponding to the C-H bond of methyl (–CH_3_) and methylene (CH_2_–) groups of Aliquat 336. Additionally, bending absorption bands appear at 1600–1300 cm^− 1^ assigned to the same group [41, 43]. The characteristic quaternary amine, Aliquat 336, absorption bands at 1143.55 cm^− 1^ and 1468.04 cm^− 1^ correspond to the symmetric stretching vibrations of –C–N and –N–CH_3_, respectively [19]. Furthermore, the spectra show a broad absorption band at 3374.90 cm^− 1^ that can be corresponded to the OH vibrational stretching group of water dissolved in the quaternary amine [41]. It was observed that the intensity of the absorption bands of the–C–N and –N–CH_3_ bonds in the loaded Aliquat 336 significantly decreases when compared with the pure organic. Furthermore, the absorption band of the –C–N bond was slightly shifted from 1143.55 cm^to 1^ to 1145.26 cm^–1^. These observations prove the assumption that the selenium(IV) extraction mechanism is achieved by addition reaction, as in Eq. (5) [41].

#### Loading capacity

**Fig. 6:**
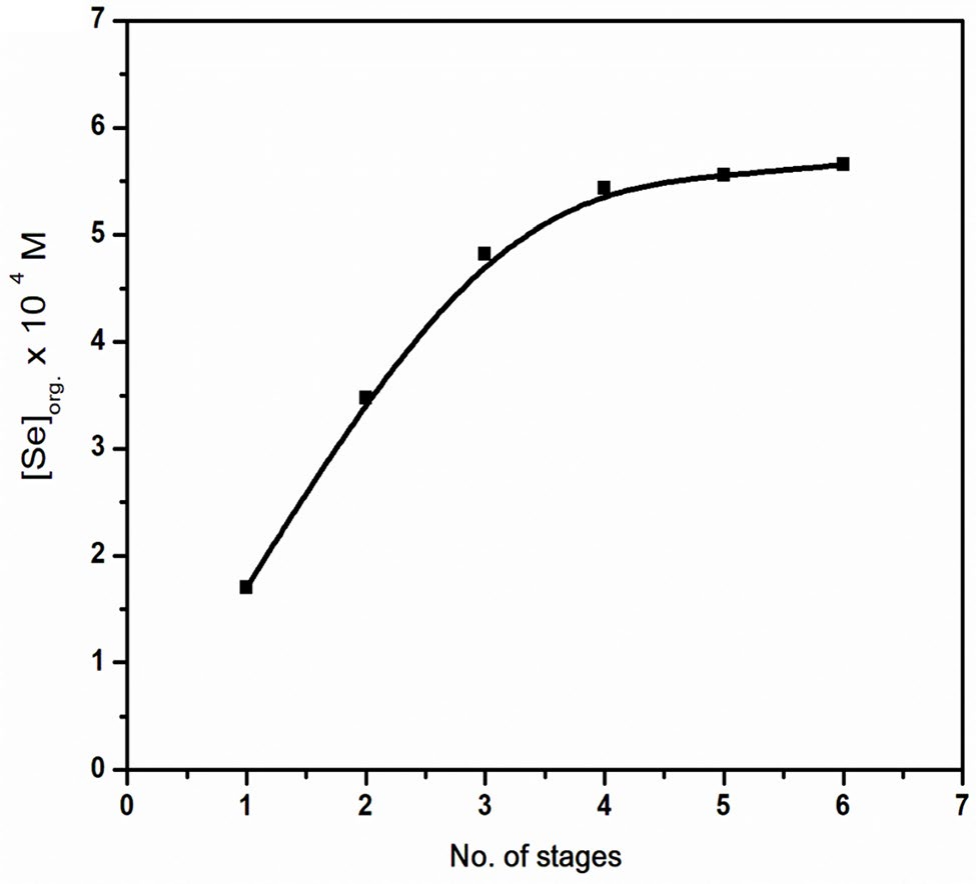
The loading capacity of the extractant as function of the effect of number of stages on the extraction of selenium(IV) with Aliquat 336/kerosene. [Aliquat 336] = 0.4 mol/L; t = 45 min; [HCl] = 6 mol/L; T = 25 ^o^C; O: A = 1:1

The loading capacity of the extractant (Aliquat 336) as a function of the effect of the number of stages on the extraction of selenium(IV) with Aliquat 336/kerosene has been investigated. By sequentially mixing fresh aqueous phase solution containing 6.3 × 10^− 4^ mol/L selenium(IV) in 6 mol/L HCl with the same organic phase containing 0.4 mol/L Aliquat 336 in kerosene-based solution at a fixed phase ratio, O:A = 1:1, the loading capacity of Aliquat 336 was assessed. After each phase separation, the amount of selenium(IV) in the aqueous phase was measured until no more selenium(IV) extraction was noticed. After 6 extraction stages, a maximum selenium(IV) concentration of 5.65 × 10^− 4^ mol/L was found in the organic phase, as shown in Fig. 6. Consequently, this means that the maximum loading of Aliquat 336 for the extraction of selenium(IV) from 6 mol/L HCl is 1.41 × 10^− 3^ mol/L selenium(IV) per mole of extractant.

#### Effect of phase ratio

**Fig. 7:**
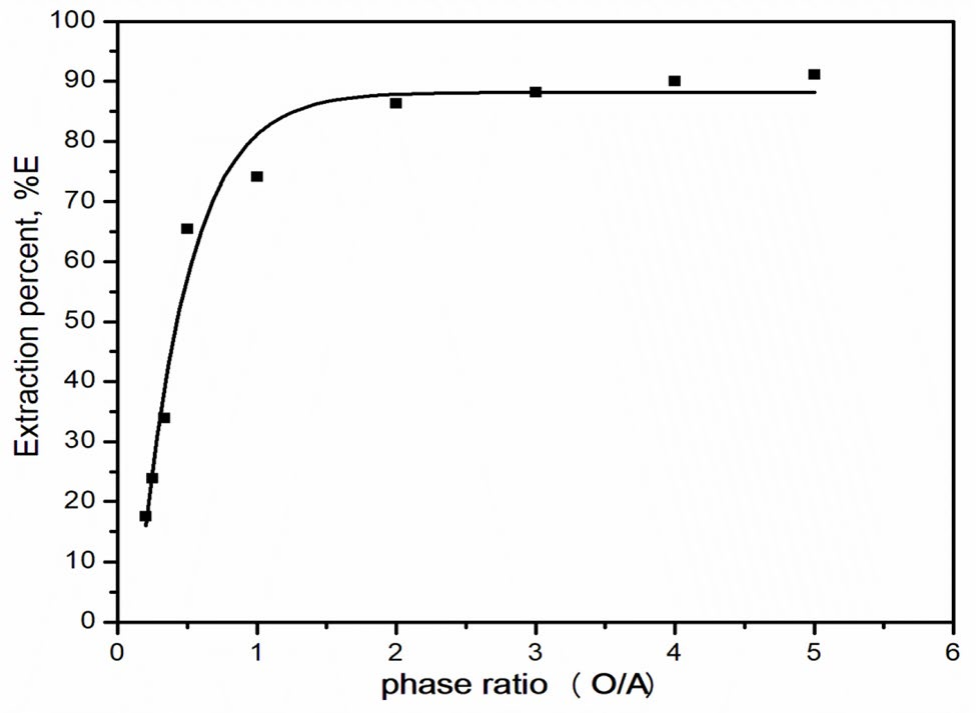
Effect of organic to aqueous phase ratio on the extraction of selenium(IV) using Aliquat 336 / kerosene. [Aliquat 336] = 0.4 mol/L; t = 45 min; [HCl] = 6 mol/L; T = 25 ^o^C; [Se(IV)] = 0.05 g/L

Results of contacting different volume ratios of organic to aqueous phase at various O/A phase ratios of 1:5 and alternatively from 5:1 were used to perform the extraction and are presented in Fig. 7. The data indicate that a preferred O/A phase ratio exists for the system under study, and this was found to be 1:1. This is evident from the sharp increase in the distribution coefficient of selenium(IV) when the phase ratio changed from 1:5 to 1:1. This may simply be due to the unavailability of reagents for metal extraction, and so a crowding effect occurs at low phase ratios.

#### Effect of temperature

**Fig. 8:**
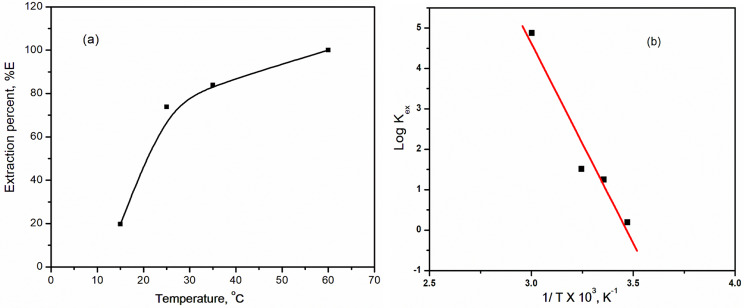
**(a)** Influence of temperature on the extraction of selenium(IV) from high acidic concentration **(b)** Relation between K_ex_ and 1/T in K^o^. [Aliquat 336] = 0.4 mol/L; t = 45 min; [HCl] = 6 mol/L; T = 25 ^o^C; [Se(IV)] = 0.05 g/L

The influence of temperature on the extraction of selenium(IV) from an aqueous hydrochloric acid solution using Aliquat 336 in a kerosene-based solution was studied within the temperature range 15–60 ^o^C. The data shown in Fig. 8a illustrates that the selenium(IV) extraction increased from ~ 25% to ~ 98% with an increase in temperature from 15 to 60 ^o^C, indicating the endothermic nature of the extraction reaction according to the positive value obtained of the enthalpy change(ΔH). The explanation of this behavior may be related to the high viscous properties of Aliquate 336 that hinder the extractability of the metal ions and were overcome by increasing temperature [19]. The linear relationship between the ln K_ex_ and 1/T is presented in Fig. 8b. From the slope and the intercept of the obtained straight line, the thermodynamic parameters such as enthalpy change (ΔH), the free energy change (ΔG), and the entropy change (ΔS) were estimated using the Van’t Hoff Eqs. [44, 45].

9 $$\:{Log\:K}_{ex}=\:-\frac{\varDelta\:H}{2.303RT}+\:C$$

10 $$\:\varDelta\:G=\:-RTLn\:{K}_{ex}$$

11 $$\:\varDelta\:H=\:-\:\frac{d\left(Ln\:{K}_{ex}\right)}{d(1/T)}$$

12 $$\:\varDelta\:S=\:\frac{\varDelta\:H-\:\varDelta\:G}{T}$$

where R denotes the universal gas constant (8.314 J mol^− 1^ K^− 1^), T refers to the absolute temperature, and C is a thermodynamic constant. Thermodynamic parameters (ΔH), (ΔG), and (ΔS) were computed for the extraction of selenium(IV) and are presented in (Table 1). The positive sign of ΔH (9884.14 kJ/mol) value indicates the endothermic nature of the selenium(IV) extraction with Aliquat 336, and the negative sign of ΔG (-7112.47 kJ/mol) value is an indication.

**Table 1:** Thermodynamic parameter values for the extraction of selenium(IV) from 6 mol/L HCl solution by Aliquat 336/kerosene

Thermodynamic parameter	Calculated value
Enthalpy change (ΔH)	82.18 k J mol^− 1^
Free energy change (ΔG)	-7.112 k J mol^− 1^
Entropy change (ΔS)	299.6 J mol^− 1^K^− 1^

Of the spontaneous nature of the extraction process. The positive value of the entropy change ΔS (57.04 J/mol) in the case of using Aliquat 336 indicates the increase in the disorder of the investigated system.

#### Effect of diluents

**Fig. 9:**
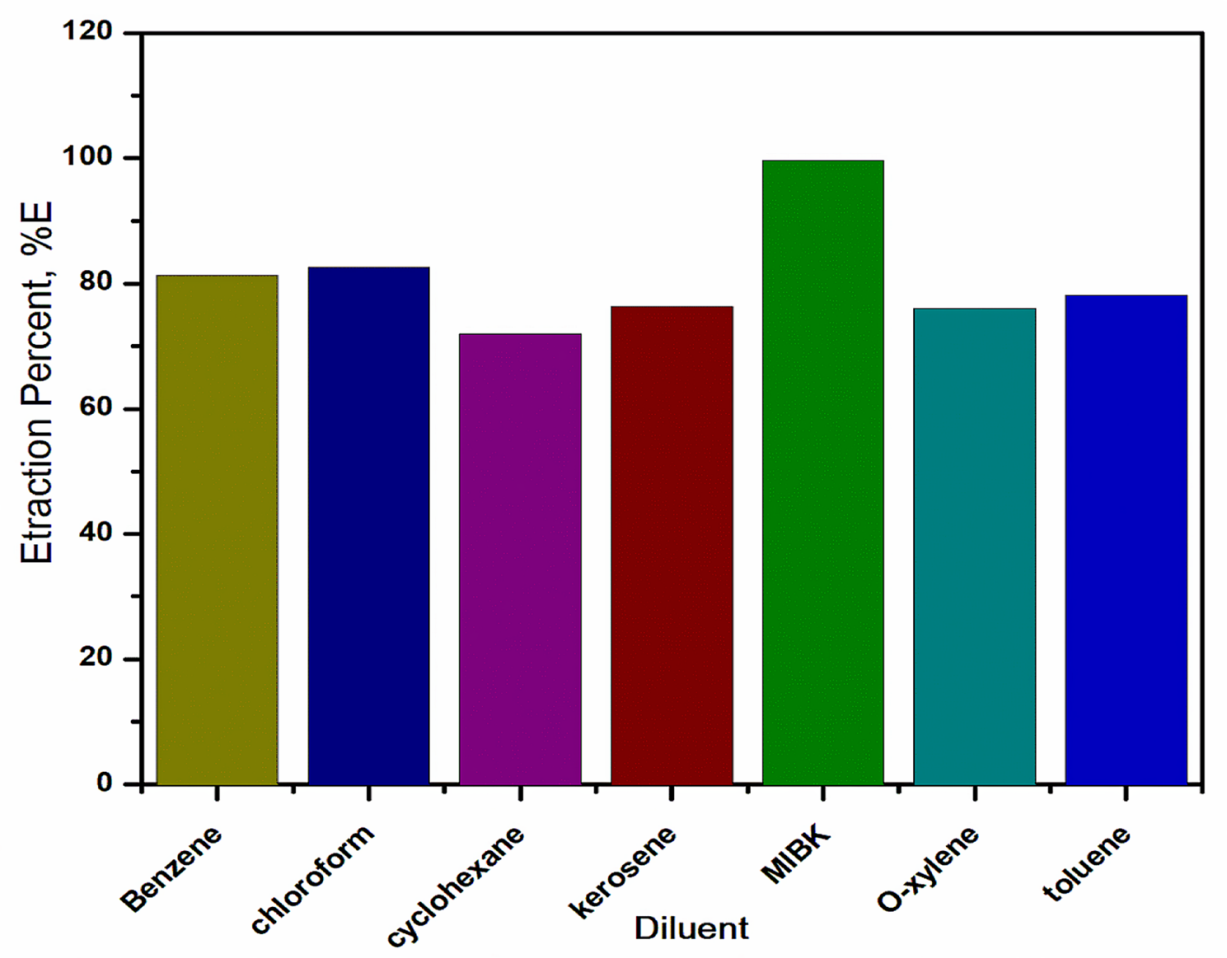
Effect of various diluents on selenium(IV) extraction from HCl solution with Aliquat 336 at O: A = 1. [Aliquat 336] = 0.4 mol/L; Shaking time = 45 min; [HCl] = 6 mol/L; T = 25 ^o^C; [Se(IV)] = 0.05 g/L

Different diluents were tested for the extraction efficiency of Aliquat 336 for selenium(IV) extraction from hydrochloric acid solution. This was investigated using various aliphatic and aromatic diluents. The results given in Fig. 9 indicate that kerosene, chloroform, cyclohexane, benzene, toluene, and o-xylene demonstrated efficient closer extraction percentages. On the other hand, the results show that the extraction efficiency of selenium(IV) reached its maximum extraction percentage of 99.59% when 0.4 mol/L Aliquat 336 was dissolved in methyl isobutyl ketone (MIBK). The extractability of Aliquat 336 with the studied diluents increased in the following order: cyclohexane < o-xylene < kerosene < toluene < benzene < chloroform < MIBK. This sequence order is attributed to the dielectric constant (Ɛ) and dipole moment (µ) of the studied diluents [44], as shown in Table 2. The highest %E occurring with MIBK was related to its highest dielectric constant. Additionally, this result may be attributed to the variation in the strength of the extractant – diluent interactions. The much stronger reagent-diluent interaction has the lowest extraction efficiency [46]. However, owing to safety grounds, economic considerations (the lower cost), availability, and lower toxicity of kerosene, it was selected as the most appropriate diluent for all the experiments conducted in this paper.

**Table 2:** Physical properties effect of different aromatic and aliphatic diluents on extraction of selenium(IV) with Aliquat 336 from hydrochloric acid solution

Diluent	ε	µ	%E
Benzene	2.27	0	81.23
Toluene	2.38	0.375	78.16
O-xylene	2.56	0.62	76.06
Cyclohexane	2.1	0	71.96
Chloroform	4.81	1.15	82.65
Kerosene	2.002	0	76.26
MIBK	13.11	2.8	99.59

%E extraction percentage of selenium(IV); µ dipole moment; ε dielectric constant

#### 11. McCabe-Thiele Diagram

**Fig. 10:**
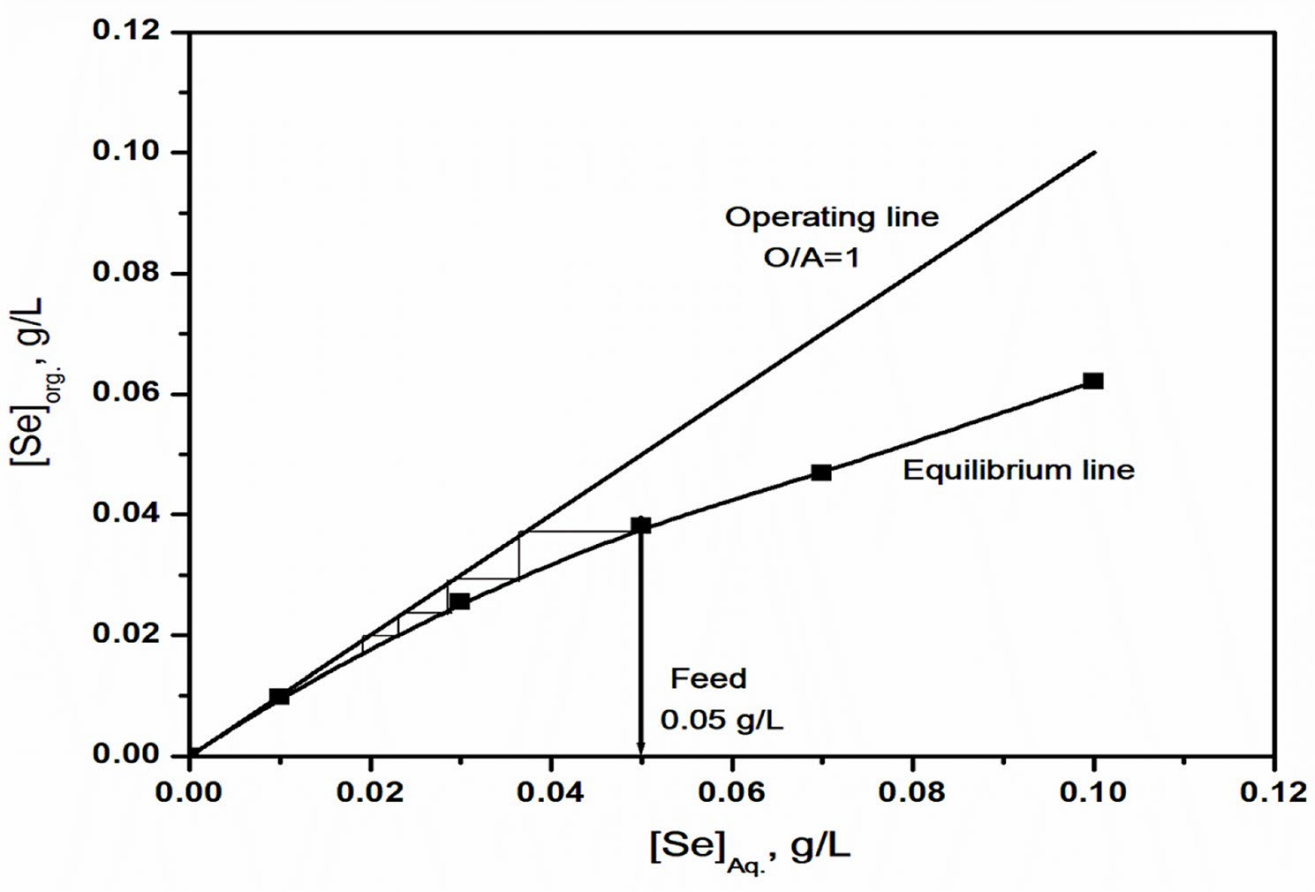
McCabe–Thiele diagram for theoretical stages of selenium(IV) extraction from HCl solution with Aliquat 336/Kerosene at 25 °C and O: A = 1:1

The number of the theoretical stages required for achieving the complete extraction of 0.05 g/L selenium(IV) from 6 mol/L HCl solution with 0.4 mol/L Aliquat 336 in kerosene at O/A = 1:1 could be portended using the McCabe-Thiele diagram, Fig. 10. To create the diagram, first draw the extraction isotherm (named equilibrium line) that describes the relationship between selenium(IV) concentration in the aqueous phase and selenium(IV) concentration in the organic phase. After that, a vertical line is drawn on the X-axis from the concentration of selenium(IV) in the feed solution. Additionally, an operating line is plotted, whose slope is equal to the phase ratio (O/A = 1:1) to be used. Finally, starting from the point where the vertical line from selenium(IV) content of the feed intersects the extraction isotherm, draw a horizontal line to the operating line and then the vertical line to the equilibrium line. According to this schematic diagram, 0.4 mol/L Aliquat 336 can completely extract selenium(IV) sufficiently in four extraction stages.

### Stripping investigations

The loaded organic phase containing selenium(IV) after extraction with 0.4 mol/L Aliquat 336 in kerosene-based solution was re-extracted using various concentrations of HNO_3_, H_2_SO_4_, HCl, citric acid, NaOH, as well as NH_4_OH at an equal phase ratio of 1:1, and shaking time was chosen to be 60 min to ensure complete stripping of selenium(IV) into the aqueous phase to find the suitable stripping agent. The results of the metal concentration analysis of the aqueous phase are represented in Table 3 as a relationship between the stripping percentage of selenium(IV) and the stripping agent concentrations. In general, it is challenging to completely strip the selenium(IV) ions. From the perspective of environmental safety, the extractant’s ability to hold the selenium ions in our work was excellent, which is seen as a positive outcome. The tabulated results show that 7 mol/L HNO_3_ was more effective than the other reagents owing to its ability to strip selenium(IV) successfully with about 60% in one stripping stage. Thus, the complete stripping of extracted organic species according to results could be achieved after two successive stripping stages so that the organic phase could be reused and regenerated.

**Table 3:** Stripping of selenium(IV) with different concentrations of various stripping agents after its extraction with 0.4 mol/L aliquat 336 in kerosene at an O/A phase ratio of 1:1 and at 25 °C

Stripping agent	Stripping percentage, %
HCl, mol/L	
0.1	3.15
7	2.52
HNO_3_, mol/L	
0.1	5.66
1	10.063
4	24
7	**60**
H_2_SO_4_, mol/L	
0.1 2.0	2.52 0.63
NaOH, mol/L	
0.1	2.52
0.5	10.063
1	9
Thiourea, mol/L 0.5 1	6.3 10.06
K_2_S_2_O_7_, mol/L 0.5 1	12.58 28.3
Ammonia ratio	
1:5	6.29

### Application study

The separation of selenium from copper anode slime was investigated by Pasdar et al. [28], consisting mainly of gold, silver, selenium, and tellurium. It was reported that the optimum conditions for selenium recovery by alkaline roasting were carried out at a temperature of 600 ^o^C and a time of 8 h. They indicated that about 98% selenium was recovered by the mentioned method. In this concern, the influence of diverse ions that may be contained in the leach liquor of copper anode slime on the extraction selenium(IV) in 6 mol/L HCl was investigated by adding different amounts of selected ionic species (Se^4+^, Te^4+^, Au^3+^, Cu^2+^, Ni^2+^, Pb^2+^, Zn^2+^). The amount of each ionic species added to the extraction system before extraction (taken amount) and after extraction (determined amount) was examined. The data shown in Table 4 indicate that tellurium and gold ions were mostly interfere with the extraction of selenium ions in 6 mol/L HCl with 0.4 mol/L Aliquat336 in kerosene at 1: 1 O/A ratio, which may be due to the formation of extractable chloric-species and a great similarity between tellurium and selenium in physical and chemical properties [47], whereas other common ions such as lead and nickel did not interfere alongside with selenium extraction due to the existence of non-extractable species at higher hydrochloric acid concentrations [48]. The presented study indicates that selenium (IV) could be efficiently separated from its mixture with lead and nickel using 0.4 mol/L Aliquat 336/kerosene, compared to its mixture with trillium and gold. The lowering in the extraction percentage value, %E for selenium(IV), may be due to the difference in experimental conditions in addition to the molar ratio of ions existing in the copper anode slime simulated solution. Additionally, it may be related to the competition between selenium ions and ions of the other metals in the simulated solution. The results indicated that selenium(IV) was preferred extracted from a binary mixture of nickel and lead in 6 mol/L HCl using 0.4 mol/L Aliquat 336/kerosene. Additionally, the extraction system of 6 mol/L HCl (aqueous phase) / 0.4 mol/L Aliquate 336 in kerosene-based solution (organic phase) is the best system for complete extraction of tellurium and gold ions.

**Table 4:** Extraction of selenium(IV) from mixture of metal ions (Se^4+^, Te^4+^, Au^3+^, Cu^2+^, Ni^2+^, Pb^2+^, Zn^2+^) that may be contained in the leach liquor of copper anode slime dissolved in 6 mol/L HCl by 0.4 mol/L aliquat 336/kerosene

*Ions*	*Amount Taken* *mg/L*	*Amount determined* *mg/L*	*%E*
Se(IV) Te(IV) Au(III) Ag(I) Cu(II) Ni(II) Pb(II) Zn(II)	50.00 11.40 27.55 75.89 179.1 10.81 163.2 1.900	*32.2* 0.00 0.00 34.0 *63.9* *10.81* 163.43 0.600	*35.6* *≥ 99* ***≥99*** *55.2* *61.55* ***0.00*** ***0.00*** *68.4*

## Conclusion

In summary, this research focus on the investigations of the extraction efficiency of ionic liquid, Aliquat 336 dissolved in kerosene diluents, for extraction of selenium(IV) from hydrochloric acid solution. To sum up, our findings indicate that the extractability of selenium (IV) was dependent on the acidity and extractant concentration. The extraction of selenium (IV) is low value at low concentrations of the acid and extractant, reaching approximately 3.4% at 1 mol/L HCl and 39.5% at 0.1 mol/L extractant, respectively, and it increases with the increase in both the acidity and extractant concentrations, reaching approximately 92% at 8 mol/L HCl and 97.1% at 1 mol/L extractant, respectively. Selenium(IV) was extracted quantitatively with 0.4 mol/L Aliquat 336 in kerosene after 45 min, 6 mol/L HCl, O:A = 1:1, and at 25 oC. Based on the experimental findings and using the slope analysis method, the extracted species was found to be [H2SeO2Cl2.2R4NCl]org that was confirmed by FT-IR analysis. Kerosene is recommended to be the most preferred diluent compared with other investigated aliphatic and aromatic diluents. After extraction under the above specified circumstances, nitric acid was found to be the most efficient stripping agent (~ 60%). Selenium(IV) could be recovered quantitatively from a synthetic mixture of different cations that are found in the leach liquor of copper anode slime by the proposed method. The current study offers a straightforward technique for the quantitative recovery of selenium(IV) from the simulated solution of copper anode slime. Furthermore, the most recommended system for fully extracting tellurium and gold ions is 6 mol/L hydrochloric acid medium (aqueous phase) using 0.4 mol/L Aliquate 336 in kerosene-based solution (organic phase).

## Data Availability

The datasets used and/or analysed during the current study are available from the corresponding author on reasonable request.

## Abbreviations

C_o_: Concentration of metal ion in the aqueous phase before extraction
C: Concentration of metal ion in the aqueous phase after extraction
D: Distribution coefficient
%E: Extraction percentage
C_s_: Concentration of metal ion in the aqueous phase after stripping
CYANEX 925: Tri-alkylphosphine oxide
HDEHP: Di(2-ethylhexyl)phosphoric acid
HTTA: Thenoyltrifluoroacetone
O: A: Organic : Aqueous phase ratio
K_ex_: Equilibrium constant
R: Universal gas constant (8.314 J mol^− 1^ K^− 1^)
T: Temperature in Kelvin
ΔG: Free energy change
ΔS: Entropy change
ΔH: Enthalpy change
µ: Dipole moment
ε: Dielectric constant
MIBK: Methyl isobutyl ketone
